# Supplementary material for: Down Regulation of Catsper1 Expression by Calmodulin Inhibitor (Calmidazolium): Possible Implications for Fertility
Source: Int J Mol Sci. 2022 Jul 22;23(15):8070. doi: 10.3390/ijms23158070 (PMC9331981; doi:10.3390/ijms23158070)
Supplement: Supplementary file 1 [file ijms-23-08070-s001.zip › ijms-1809575-supplementary.pdf]

International Journal of
Molecular Sciences

MDPI

Supplemental Material

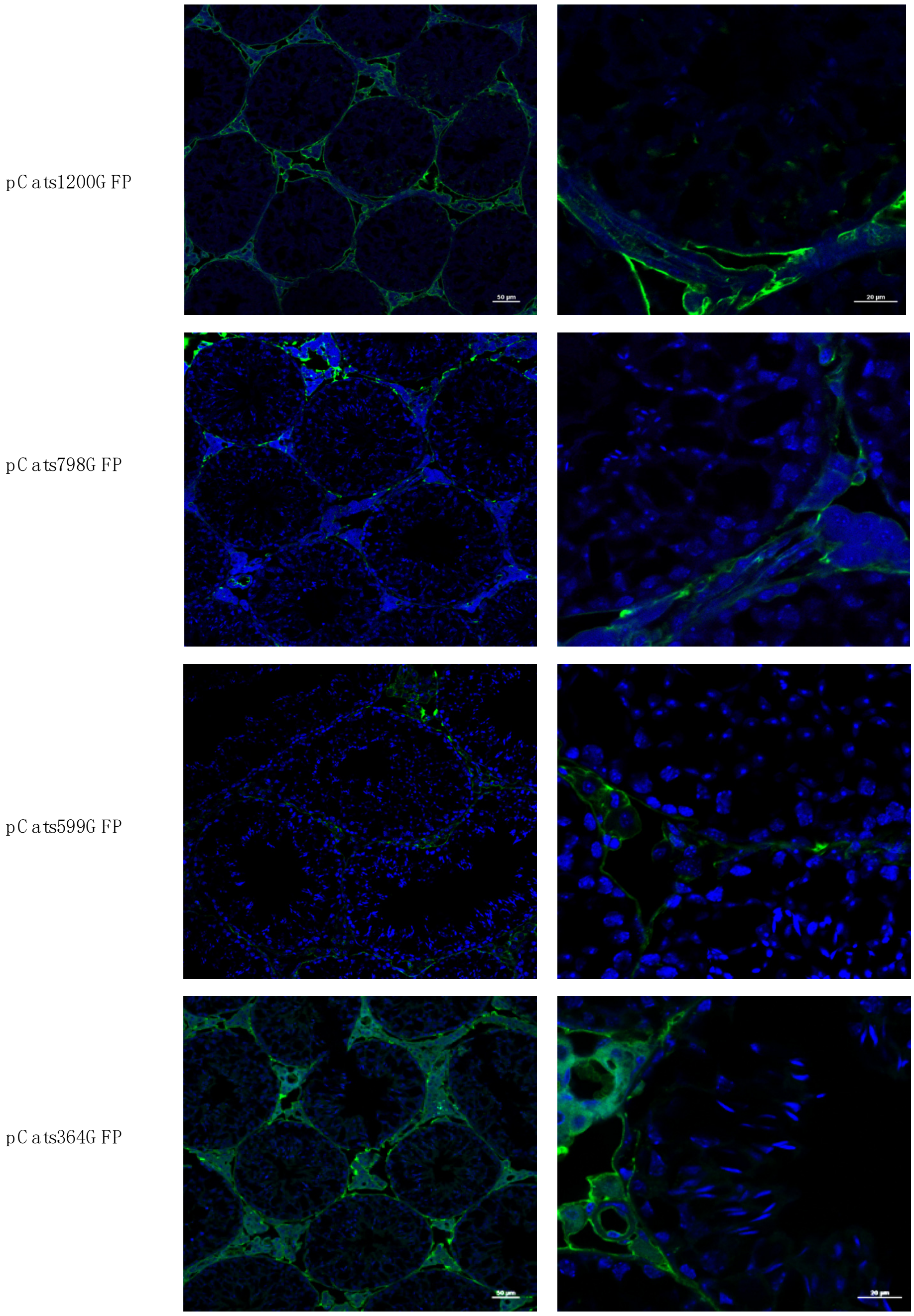

Figure S1. In vivo Sox5 immunodetection in murine testes transfected with the *Catsper1* promoter. The constructs with different deletions of the *Catsper1* promoter and containing the GFP gene as a reporter gene of transcription were transfected by intratesticular injection in mice. The left column shows Sox5 immunodetection with an anti-mouse antibody coupled to Alexa 486 with a 20×magnification. The right column shows images with 60×magnification. Names of the constructs are aside from each pair of photos. The green signal corresponds to Sox5 and is observed in the cytoplasm of interstitial cells, only pCats1200GFP showed a weak signal in germ cells. DAPI was used to contrast the cell nuclei.

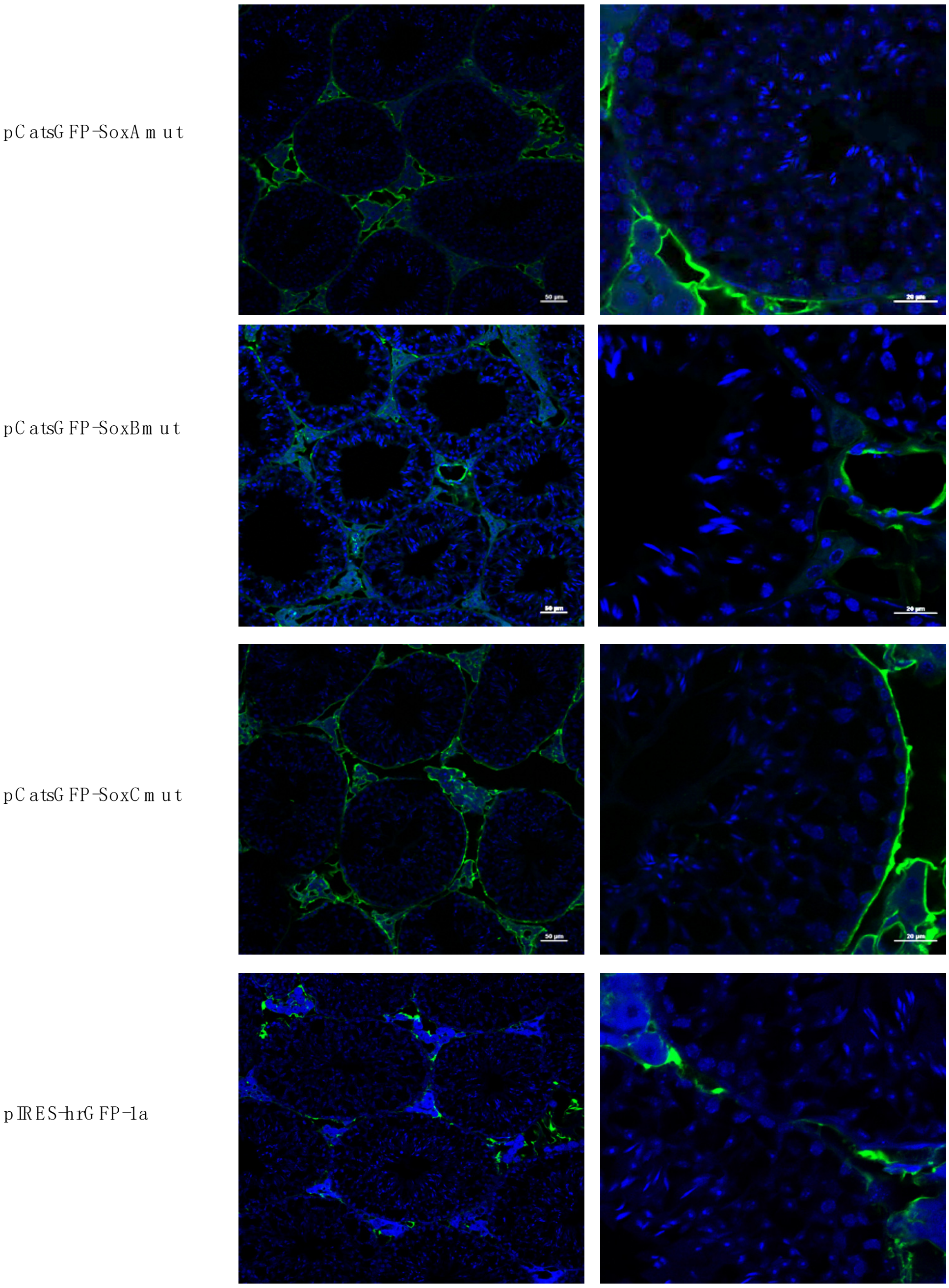

Figure S2. Sox5 expression from plasmids with Sox mutations *Catsper1* promoter in vivo. The constructs with Sox A, B, or C mutations of the *Catsper1* promoter and containing the GFP gene as a reporter gene for transcription, were transfected by intratesticular injection in mice. The left column shows 10 µm sections exposed to the Sox5 antibody and revealed with an anti-mouse secondary antibody coupled to Alexa 486, preparations were observed in a confocal microscope with a 20×magnification. The right column shows endogenous Sox5 with a 60× magnification. A green signal is observed in the cytoplasm of somatic cells of the seminiferous tubules. DAPI was used to contrast the cell nuclei.
